# A Community Detection Approach to Cleaning Extremely Large Face Database

**DOI:** 10.1155/2018/4512473

**Published:** 2018-04-22

**Authors:** Chi Jin, Ruochun Jin, Kai Chen, Yong Dou

**Affiliations:** ^1^Computer School, University of South China, Hengyang, China; ^2^National Laboratory for Parallel and Distributed Processing, National University of Defense Technology, Changsha, China

## Abstract

Though it has been easier to build large face datasets by collecting images from the Internet in this Big Data era, the time-consuming manual annotation process prevents researchers from constructing larger ones, which makes the automatic cleaning of noisy labels highly desirable. However, identifying mislabeled faces by machine is quite challenging because the diversity of a person's face images that are captured wildly at all ages is extraordinarily rich. In view of this, we propose a graph-based cleaning method that mainly employs the community detection algorithm and deep CNN models to delete mislabeled images. As the diversity of faces is preserved in multiple large communities, our cleaning results have both high cleanness and rich data diversity. With our method, we clean the extremely large MS-Celeb-1M face dataset (approximately 10 million images with noisy labels) and obtain a clean version of it called C-MS-Celeb (6,464,018 images of 94,682 celebrities). By training a single-net model using our C-MS-Celeb dataset, without fine-tuning, we achieve 99.67% at Equal Error Rate on the LFW face recognition benchmark, which is comparable to other state-of-the-art results. This demonstrates the data cleaning positive effects on the model training. To the best of our knowledge, our C-MS-Celeb is the largest clean face dataset that is publicly available so far, which will benefit face recognition researchers.

## 1. Introduction

In the last few years, researchers have witnessed the remarkable progress in face recognition due to the significant success of deep convolutional neural networks [1] and the emergence of large scale face datasets [2]. Although the data explosion has made it easier to build datasets by collecting real world images from the Internet [3], constructing a large scale face dataset remains a highly time-consuming and costly task because the mislabeled images returned by search engines need to be manually removed [4]. Thus, automatic cleaning of noisy labels in the raw dataset is strongly desirable.

However, identifying mislabeled faces automatically by machine is by no means easy. The main reason for this is that, for faces that are captured wildly, the variation of a man's faces can be so large that some of his images may easily be identified as someone else's [5]. Thus, a machine may be misled by this rich data diversity within one person and delete correctly labeled images. For example, if old faces of a man are the majority in the dataset, a young face of him may be regarded as someone else and removed. Another challenge is that, due to the ambiguity of people's names, searching for someone's pictures online usually returns images from multiple people [2], which requires the cleaning method to be tolerant to the high proportion of noisy labels in the raw dataset constructed by online searching.

In order to clean noisy labels and meanwhile preserve the rich data diversity of various faces, we propose a three-stage graph-based method to clean large face datasets using the community detection algorithm. For each image in the raw dataset, we firstly use pretrained deep CNN models to align the face and extract a feature vector to represent each face. Secondly, for features of the same identity, based on the cosine similarity between different features, we construct an undirected graph, named “face similarity graph,” to quantify the similarity between different images. After deleting weak edges and applying the community detection algorithm, we delete mislabeled images by removing minor communities. In the last stage, we try to relabel each previously deleted image according to the similarity between its feature and the center of each community that is reserved in Stage 2. In our method, we make two assumptions that are satisfied in most cases. First, for images of the same identity, the visual similarity between mislabeled ones is relatively weak. Secondly, most mislabeled images returned by the search engine are usually other celebrities' faces because the online top query results are always celebrities, which makes the relabeling stage possible.

Theoretical analysis and extensive experiments demonstrate that our method is able to delete almost all mislabeled images and preserve rich diversity of each person's different faces, which benefits the generalization of face recognition models [4]. With one desktop PC, it took us 76 hours to obtain a clean version of the MS-Celeb-1M dataset [2] called C-MS-Celeb (containing 6,464,016 face images of 94,682 celebrities) and approximately 97.3% of images in it are correctly labeled. Using our C-MS-Celeb dataset, without fine-tuning, we train an Inception-ResNet-v1 model [6] and achieve state-of-the-art results on the LFW face recognition benchmarks. The Equal Error Rate of the model trained with our C-MS-Celeb is 99.67%, 0.27% higher than that of the model trained using the original MS-Celeb-1M, which proves the data cleaning positive effects on the model training. To the best of our knowledge, our C-MS-Celeb is the largest clean face dataset that is publicly available so far, which will benefit face recognition researchers, especially in academia.

## 2. Related Work

### 2.1. Face Dataset Cleaning

Previous work can be divided into two categories which are the data-driven approach and the human assisted approach.

Without employing human annotators, the data-driven approach utilizes search engines, face recognition models, and outlier detection methods to remove noisy labels. Specifically, the authors of [7] formulated the cleaning task as a quadratic programming problem and solved it with optimization methods. In [8], an anchor face that had the most neighbors was selected and a maximal subgraph starting from this anchor was regarded as the cleaning result. However, due to their limited noise tolerance, most previous solutions make the assumption that the proportion of mislabeled images is small (less than 20%). Another limitation of previous work is that the cleaning results lack diversity because peculiar images with unusual poses, expressions, or illumination may well be regarded as outliers and deleted [8]. Though [9] has proposed a semantic bootstrapping method to cleaning the noisy MS-Celeb-1M, each identity is represented by only one center in the softmax loss function, which lowers the diversity of images in their cleaning result.

By employing annotators, the human assisted approach has succeeded in constructing a variety of face datasets where most images are correctly labeled [10, 11]. However, due to the limitation of labour force and time, manually labeling extremely large datasets is nearly impossible. Though authors of [12] have proposed an active annotation and learning framework combining human annotators with deep learning models to clean noisy labels in large datasets, the iterative human annotation in this process is still time-consuming.

### 2.2. MS-Celeb-1M Face Dataset

Released by Microsoft Research in 2016, MS-Celeb-1M [2] has been the largest face dataset (8,456,240 images of 99,892 celebrities) that is publicly available. However, without data cleaning that was left by the dataset maker as an open question, a considerable percentage of images are mislabeled because all images are query results directly returned by search engines. This may have detrimental influence on supervised learning [13].

### 2.3. Community Detection in Graphs

Community detection is a topic in graph theory research, aiming at extracting the community structure of graphs [14]. Though extensive research has been done in this field, the potential benefits of community detection to the machine learning community have long been ignored [15].

## 3. Method

### 3.1. Notation and Problem Statement

Given a face dataset *F* which includes *N* subjects, we can divide it into *N* subsets based on the identity label and images in each subset *F*^*i*^ are labeled with *α*^*i*^. With deep CNN models, we can extract a set of feature vectors *Ω*^*i*^ for each face *f*^*ij*^ in *F*^*i*^ and each feature vector is denoted as *ω*^*ij*^ ∈ *Ω*^*i*^, *j* = 1,…, |*Ω*^*i*^|, where |*Ω*^*i*^| is the cardinality of the set *Ω*^*i*^. ‖*ω*‖_2_ represents the L2 norm of *ω* and *Ω* = ⋃_*i*=1_^*N*^*Ω*^*i*^ denotes the set of features from all *N* subjects in the dataset. The aim of the face dataset cleaning task is to remove mislabeled images and meanwhile preserve correctly labeled ones. In this paper, we focus only on the identity label and ignore other annotations such as poses, bounding boxes, and gender. The phrase “images of the same identity” means images that have the same identity label.

### 3.2. Method Overview

**Figure 1:**
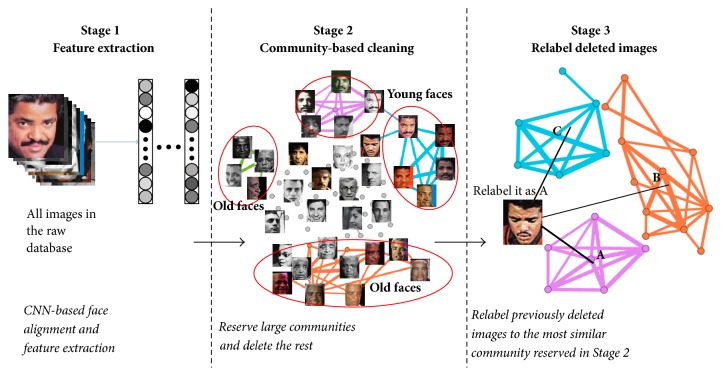
After feature extraction, we delete mislabeled images by detecting communities in the “face similarity graph,” reserving large communities and removing the rest. Finally, we relabel the previously deleted images.

Our cleaning method consists of three main stages (Figure 1). First, all images in the raw dataset are sent to the pretrained deep CNN model and a 128D feature vector is extracted to represent each image. Then, for features of the same identity, we construct a “face similarity graph” based on the cosine similarity between different features. After deleting weak edges in the graph and employing the community detection algorithm, we reserve the relatively large communities and delete the rest. Finally, each previously deleted image has a chance to gain a new identity label and return to the dataset according to the similarity between its feature and the center of each community reserved in Stage 2.

### 3.3. Stage 1: Face Alignment and Feature Extraction

Deep learning approaches have achieved remarkable performance on face detection, alignment, and feature extraction [16]. Thus, in the first stage, we employ pretrained deep CNN models to preprocess faces and extract features. Given an image *f*^*ij*^, we firstly utilize the multitask cascaded convolutional networks [17] to detect and align the face inside. Then the deep CNN model in [6] is used to extract a feature vector *ω*^*ij*^ for the aligned face. After this stage, each image is represented by a 128D feature vector.

### 3.4. Stage 2: Face Similarity Graph and Community-Based Cleaning

In this section, we firstly give the definition of the face similarity graph and its construction algorithm. Then we demonstrate how to delete mislabeled images by applying the community detection algorithm to this graph.

#### 3.4.1. Definition of Face Similarity Graph

Given *n* feature vectors, each representing one face image, the face similarity graph *G* = (*V*, *E*) of these features consists of a set of vertexes *V* = {*v*_1_,…, *v*_*n*_} and undirected edges *E*⊆*V* × *V* without self-loop. Each vertex *v* ∈ *V* represents one image. The weight of the edge *e*_*j*,*k*_ ∈ *E* quantifies the similarity between image *j* and image *k* and the larger the weight *w*_*j*,*k*_ of the edge is, the stronger the similarity is.

#### 3.4.2. Construction of Face Similarity Graph

(1) $$\begin{array}{c} CosSimilarity\left(\;\omega^{ij},\omega^{ik}\;\right)=\frac{{\omega^{ij}}^{T}\cdot \omega^{ik}}{{\left\|\omega^{ij}\right\|}_{2}{\left\|\omega^{ik}\right\|}_{2}}. \end{array}$$

Using the features *Ω*^*i*^ extracted from images of the same identity, Algorithm 1 constructs the corresponding face similarity graph. For each pair of the features, we compute their cosine similarity, detailed in (1), and save the positive results as the weights of the edges. Due to the powerful expressive ability of the feature vectors extracted by the deep CNN model, the cosine similarity is able to quantify the visual similarity between two faces [16].

**Figure 2:**
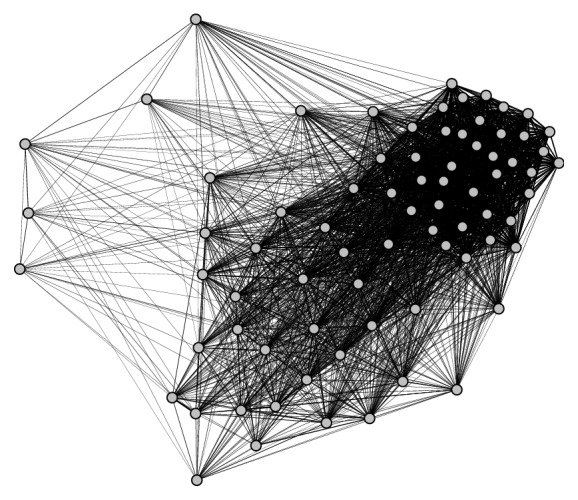
The face similarity graph of Phil Upchurch in the MS-Celeb-1M dataset. Each vertex represents an image of Phil and the weight of an edge quantifies the similarity between two images. The right part is closely connected while the left part is sparse.

For illustration purpose, the face similarity graph of Phil Upchurch, a famous American guitarist, in MS-Celeb-1M is shown in Figure 2. We depict the graph using Gephi [18] and “Force Atlas” is selected as the graph layout algorithm. As shown in Figure 2, most vertexes are closely connected by thick edges, which constitute a compact group in the right part. This compactness means that images within this group are quite visually similar, which indicates that these faces may belong to the same person. Conversely, in the left part of the graph, a handful of vertexes are loosely connected, which suggests that they are dissimilar to any other faces. These sparse vertexes probably represent mislabeled images that are dissimilar to each other.

#### 3.4.3. Community-Based Deletion of Mislabeling

In order to delete mislabeled images, we remove weak edges from the face similarity graph, apply the community detection algorithm, and reserve relatively large communities. This community-based cleaning approach is effective because the visual similarity among correctly labeled images is relatively strong while that among mislabeled images is negligible.

First, any edge whose weight is less than *τ* is removed from the face similarity graph. This makes most mislabeled images become isolated and can hardly mix with correctly labeled faces, which guarantees the high cleanness of the result. In order to determine the value of *τ*, we employ the theory of open-set face identification [19]. In the open-set identification task, a system determines if a probe corresponds to a person in the photo gallery. This scenario is similar to our cleaning task where we need to determine whether a vertex in the graph corresponds to the person in the dataset. Based on our open-set identification tests using the classical LFW benchmark [20], we adopt the threshold value when FAR = 1% and assign it to *τ*. This choice means that, statistically, only 1% of all mislabeled images will be misidentified as correctly labeled ones and sneak into large communities during the community detection later, which ensures the high cleanness of our result.

**Figure 3:**
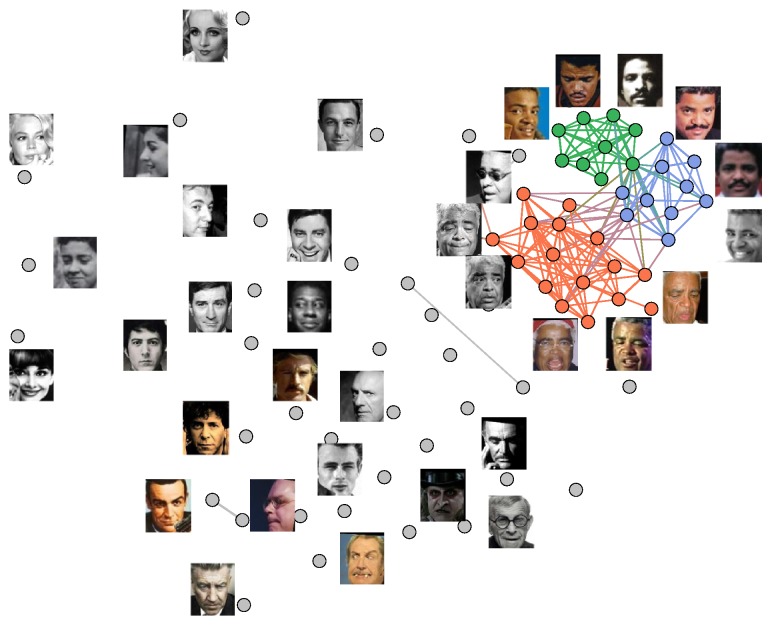
Community detection result of Phil Upchurch's face similarity graph. The green, indigo, and orange communities representing young, middle-age, and old face images of Phil are reserved while the rest are deleted.

After deleting weak edges, we apply the fast community detection algorithm [21] to reveal the community structure inside the graph. The reasons for choosing [21] are its high speed and stable detection results, which enable us to process extremely large datasets efficiently.  Figure 3 illustrates the community detection result of Figure 2 after removing the weak edges, where large communities are colorized and minor communities (mostly isolated vertexes) are in grey shade. Then a community will be deleted, if the number of vertexes within the community is less than *ρ* percent of the number of all vertexes. For example, setting *ρ* as 10, the colorized major communities in Figure 3 are reserved and the rest are deleted.

Our community-based cleaning method is able to guarantee both high cleanness and rich data diversity. The high cleanness is derived from the deletion of weak edges controlled by parameter *τ* and the removal of minor communities decided by *ρ*. Due to the deletion of weak edges, mislabeled images that are visually different from each other become isolated and can hardly form big communities in the graph. Meanwhile, however, correctly labeled faces can still stay connected because of their strong similarity, which enables them to constitute large communities. Thus, high cleanness can be obtained because mislabeled images represented by minor communities are removed and correctly labeled images within large communities are reserved. This mechanism still functions well given high percentage of noisy labels because although the proportion of mislabeling is high, there is little strong similarity among mislabeled images, which prevents them from forming large communities. Our method can also ensure rich data diversity because each celebrity is represented by multiple communities. Images within the same community tend to be similar and each community represents one subcategory of an identity. For example, in Figure 3, old, middle-age, and young images of Phil are represented by the orange, indigo, and green communities, respectively. By collecting faces from multiple fine-grained communities, the data diversity of the original dataset can be preserved. Compared with [9] where each celebrity is implicitly represented by only one center in the softmax loss function, our method is able to achieve high cleanness without sacrificing data diversity.

### 3.5. Stage 3: Relabeling Previously Deleted Images

In order to maintain the large scale of the original dataset, we try to relabel each previously deleted image based on the cosine similarity between its feature and the center of each reserved community. The prerequisites for this stage are twofold. First, most mislabeled images in the raw database should be celebrity faces as well. Secondly, the number of celebrities in the raw database should be as large as possible, which will increase the possibility of successful relabeling. These two prerequisites are satisfied when cleaning the MS-Celeb-1M because images in the original database are top query results directly returned by search engines which are usually celebrity faces. In addition, the number of celebrities in MS-Celeb-1M is sufficiently large (around 100K), including almost all celebrities in all fields.

We firstly introduce more notations. After Stage 2, the original face dataset *F* can be divided into *M* communities and a large set of deleted images *D*. Each community *C*^*i*^ has an identity label *l*^*i*^ from the raw database. All features for images in *C*^*i*^ are denoted as Ψ^*i*^ and each feature vector is denoted as *ψ*^*ij*^ ∈ Ψ^*i*^, *j* = 1,…, |Ψ^*i*^|. The feature vector for a deleted image *d*^*i*^ ∈ *D* is denoted as *δ*^*i*^ and Δ denotes all feature vectors of the deleted images (*δ*^*i*^ ∈ Δ). Each community *C*^*i*^ has a center *θ*^*i*^ which is defined as the arithmetic mean value of all features in Ψ^*i*^ (see (2)).

Algorithm 2 shows the relabeling process where each element in the output list *L* consists of the image's filename and its new identity label. The relabeling algorithm can be divided into two parts. Firstly, given a deleted image, we compute the cosine similarity between its feature and the center of each community. Then we find out the maximum similarity value (*maxSimi* in Algorithm 2) along with the identity label (*tmpLabel* in Algorithm 2) of the corresponding community. Secondly, if *maxSimi* is higher than the threshold *η*, we relabel the previously deleted image as *tmpLabel* and add it to the output list *L*. Otherwise, we fail to relabel this image and delete it permanently.

(2) $$\begin{array}{c} \theta^{i}=\frac{\sum_{j=1}^{\left|\Psi^{i}\right|}\psi^{ij}}{\left|\Psi^{i}\right|}. \end{array}$$

Similar to Stage 2, we also employ the theory of open-set face identification to determine *η* in Algorithm 2. Using the classical LFW benchmark, we choose the threshold value when FAR = 0.1%. This is because images in *D* are so noisy that a higher threshold should be set to maintain the high cleanness obtained in Stage 2. More details of the parameter selection can be found in the Experiments part.

### 3.6. Theoretical Analysis

We can estimate the upper bound of the proportion of correctly labeled images in our cleaning result through theoretical analysis. In Stage 2, after deleting weak edges controlled by parameter *τ*, a mislabeled image can still remain connected to a large community and be regarded as a correctly labeled face. The probability *P*_*τ*_ of this event is approximately equal to the FAR of the face recognition model at the threshold *τ*. Secondly, in Stage 3, a deleted image can be relabeled with a wrong identity and the probability *P*_*η*_ of this equals the FAR of the recognition model at the threshold *η*. As these two events are independent, the upper bound of the correct labeling proportion *P*_correct_bound_ in the cleaning result can be calculated by (3). In our case, *P*_*τ*_ = 1% and *P*_*η*_ = 0.1%. Thus, the percentage of correctly labeled faces in our cleaning result should be no more than 98.9%.

(3) $$\begin{array}{c} P_{correct\_bound}=\left(\;1-P_{\tau }\;\right)\left(\;1-P_{\eta }\;\right). \end{array}$$

## 4. Experiments

### 4.1. Implementation Details of Our Method

The original MS-Celeb-1M dataset, containing 8,456,240 images of 99,892 celebrities, was downloaded from its official website and the deep learning framework “Tensorflow” [22] was utilized to train the models. The implementation of all the models introduced in the Method part and their training setting are publicly available in the “facenet” project on Github (https://github.com/davidsandberg/facenet). We basically followed the default setting of the original “facenet” project during model training and data cleaning except for two modifications. The first modification was that the affine transform was used to adjust the 5 facial points extracted by [17]. With this adjustment, we transformed two eye points to be horizontal and each image was resized to 160 × 160. The second modification was that, during model training and data cleaning, intact 160 × 160 images were used as the CNN's inputs instead of being randomly cropped.

We employ the Inception-ResNet-v1 [6] as the face recognition model to evaluate our cleaning method and the reasons for this choice is twofold. Firstly, Szegedy et al. proposed an “Inception” structure in the Inception-ResNet-v1 model, which enables the model to learn multiscale features. As a result, the Top5 error rate of the Inception-ResNet-v1 model in the ILSVRC2012 image classification challenge is only 5.5%, which shows the powerful feature extraction ability of the Inception-ResNet-v1. Due to Inception-ResNet-v1's outstanding performance on image classification and image feature extraction, in order to achieve high face recognition accuracy that is comparable to other state-of-the-art results, we utilize the Inception-ResNet-v1 network structure to build our face recognition model. Secondly, the residual network structure is embedded in Inception-ResNet-v1, which can speed up the training of the deep learning model [6].

The hardware platform for data cleaning was a desktop PC equipped with an Intel i7 CPU and one NVIDIA Tesla K20c GPU. The face alignment and feature extraction were conducted with GPU acceleration and Matlab2016b was employed to complete the rest of the work. It took us approximately 70 hours to align all faces and extract their feature vectors, which was the most time-consuming stage, whereas the following two stages only took 6 hours. The training of face recognition models was conducted on servers with NVIDIA Titan X GPU acceleration.

### 4.2. Comparison with Other Cleaning Methods

By cleaning the extremely large and noisy MS-Celeb-1M, we compare our solution with four other cleaning methods which are the maximal subgraph method [8], the *K*-means clustering method, the fixed proportion removal method (https://www.microsoft.com/en-us/research/project/ms-celeb-1m-challenge-recognizing-one-million-celebrities-real-world/), and the Light CNN cleaning method [9]. The quadratic programming method [7] is not applicable to the MS-Celeb-1M because it is not tolerant of the high proportion of noisy labels inside. Hence we do not compare it with ours.

We briefly introduce each method as follows.

Maximal subgraph method (MSM): Given a face similarity graph, similar to Stage 2, edges that are weaker than *τ* are firstly removed. Then the vertex that has most neighbors is selected as the anchor point. Starting from this anchor point, a maximal subgraph of the similarity graph is constructed and all images within this subgraph are the cleaning results.

K-means clustering method (KCM): Given |*Ω*^*i*^| feature vectors, the *k*-means clustering algorithm is employed to divide them into *k* clusters. Secondly, a cluster will be removed if the number of features in the cluster is less than *ρ* percent of the number of all features. The selection of parameter *k* has to be empirical in this method.

Fixed proportion removal method (FPR): Given |*Ω*^*i*^| feature vectors, we calculate the arithmetic mean value of them as their center and the Euclidean distance of each vector to that center. Based on these distances, a fixed proportion of the images that are most distant from the center are regarded as noises and are deleted. The fixed proportion *ζ* of this method is adjustable and the setting of this parameter should depend on the proportion of noisy labels in the raw dataset.

Light CNN cleaning method (LCNN): A Light CNN network and a semantic bootstrapping method are utilized to train a face recognition model and clean the noisy MS-Celeb-1M dataset. We list their cleaning result (MS-1M-2R), available online (https://github.com/AlfredXiangWu/LightCNN), in Table 1 and compare it with ours.

Our method (ours): The pretrained face detection and alignment model [17] is publicly available online. Thus, we only trained an Inception-ResNet-v1 face recognition model *A*_1_ [6] on the original MS-Celeb-1M dataset (omitting shared celebrities in the LFW dataset) and *A*_1_'s EER was 99.4% on the LFW benchmark. *τ* and *η* were set as 0.66 and 0.72, respectively, to meet the FAR requirements in our method. In Stage 2, we obtained 238,666 communities for further relabeling.

In order to evaluate the cleaning result of each method, we propose two quantified metrics, “cleanness” and “diversity,” to capture the quality of a face dataset. As shown in (4), the cleanness of a face dataset is defined by the ratio of the number of correctly labeled images to the number of all images in the dataset and an ideal cleaning result should have high cleanness.

(4) $$\begin{array}{c} Cleanness\left(\;F\;\right)=\frac{\left|F_{c}\right|}{\left|F\right|}. \end{array}$$

Defined by (5), the diversity of a face dataset is the mean value of each subject's feature variance. Before using this equation, each feature vector *ω* has been divided by its own L2 norm to normalize features of different images (denoted as *ω*_normed_). This metric quantifies the variation of each person's face images in terms of illumination, pose, age, expression, and so on. The diversity of an ideal face dataset should be relatively high, which is beneficial for training a robust face recognition model. Note that more mislabeled images may also increase the diversity. Hence the quality of a face dataset can never be judged by its diversity only.

(5) DiversityF=∑i=1N∑j=1Ωiωnormedij−ωnormedi¯2/ΩiN.

The experiment results are shown in Table 1. The diversity can be automatically calculated by programmes. As for the cleanness, we randomly selected 2,500 images from each result and obtained the number of correctly labeled images by manual checking. The number of subjects and the number of images are also listed in the table. “None” at the bottom of the table represents the original MS-Celeb-1M dataset.

As is shown in Table 1, our method is able to remove almost all mislabeled images and meanwhile preserve the diversity of various faces. This is because each celebrity is represented by multiple communities, which provides fine-grained representations for each person, and the high diversity is embedded in different communities of the same identity. Though the cleanness of the MSM's result is slightly higher than ours, the diversity of its images is much lower because it only reserves the maximal subgraph which can not represent different styles of faces. Similarly, the KCM can hardly obtain high cleanness without sacrificing diversity. In addition, another significant challenge of KCM is the parameter selection. In general, the cleanness of the KCM's result rises as *k* or *ρ* increases. However, determining an appropriate combination of *k* and *ρ* is empirical and time-consuming. The main drawback of FPR is that there exists a cleanness ceiling in this method because if the proportion of noisy labels is too high, there will always be a certain number of mislabeled images closely around the center, which can not be eliminated by deleting images that are distant from the center. Although the LCNN's result has higher cleanness, the image number and the subject number of their cleaning result are far smaller than ours, which decreases the diversity of their images.

### 4.3. Benefits of Data Cleaning to Model Training

The data cleaning's positive influence on the model training is verified in this subsection. Our first-round cleaning result (bold line in Table 1) is named as “MS-C-1” and the first-round result without the Stage 3 relabeling is denoted as “MS-C-1-noR” (containing 4,269,900 images of 91,180 celebrities). Without fine-tuning, we further train another three Inception-ResNet-v1 face recognition models *A*_2_, *A*_3_, and *B*_1_ [6] with the same training setting using three different datasets that are “MS-C-1-noR,” “MS-C-1,” and “LCNN result” [9], respectively. Then the model *A*_3_ is employed to perform a second-round cleaning of the original MS-Celeb-1M database, with *τ* and *η* set as 0.51 and 0.57, respectively, to meet the FAR requirements. The parameter *ρ* is still set as 10 for tradeoff between cleanness and diversity. Named as “C-MS-Celeb,” the second-round cleaning result contains 6,464,016 images of 94,682 celebrities. Finally, we train an Inception-ResNet-v1 model *A*_4_ using our C-MS-Celeb dataset. Celebrities included in both MS-Celeb-1M and LFW datasets [20] are omitted during the training of all models. The benchmark dataset along with testing protocol for each model is the commonly used LFW dataset and the performance of each model is shown in Table 2. The performance of *A*_2_ is significantly better than that of *A*_1_, which demonstrates the positive effects of the community-based cleaning in Stage 2 on the model training. Similarly, by comparing the performance of *A*_2_ and *A*_3_, we can conclude that the relabeling of deleted images in Stage 3 which provides larger amount of correctly labeled training data is also beneficial for increasing the model's recognition ability. Model *A*_4_ trained with the C-MS-Celeb database outperforms others in all items, which shows the benefits of our incremental way of data cleaning. In addition, the accuracy of *A*_4_ trained with our C-MS-Celeb dataset is higher than that of *B*_1_ trained with “LCNN result” because our C-MS-Celeb has larger amount of clean data with higher data diversity.

**Figure 4:**
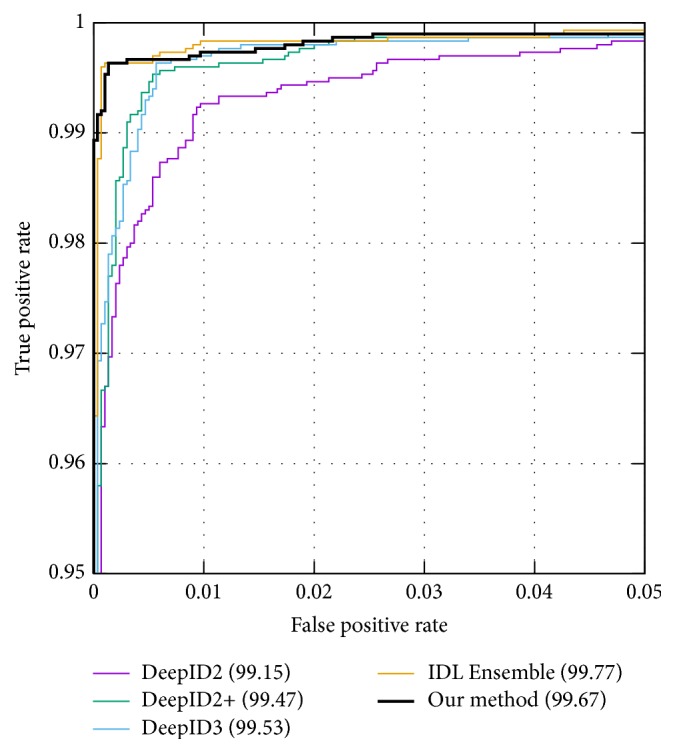
The ROC curves on the LFW benchmark.

Tables 3 and 4 compare the performance of our model with other state-of-the-art results on the LFW and LFW BLUFR [23] benchmarks. The ROC curves of top results are illustrated in Figure 4. For the LFW testing protocol, we compare our method with DeepID2+ [24], Face++ [25], DeepID3 [1], IDL Single/Ensemble Model [26], DeepFace [27], WebFace [4], Facebook-WST Fusion [28], CenterLoss [16], Light CNN-29 [9], and FaceNet [29]. Among all these methods, only IDL Ensemble Model outperforms ours in terms of “Acc” and “DIR@FAR = 1%/0.01%.” Our method is better than any other single-net model in each item. As for the LFW BLUFR benchmark, we compare ours with HighDimLBP [23], WebFace, CenterLoss, and Light CNN-29. From Table 4, we can see that our method performs the best in all LFW BLUFR tests.

Based on these experiments, we can conclude that our data cleaning does have positive effects on the model training and larger amount of clean data with rich data diversity is vital for increasing the model's recognition ability.

## 5. Conclusion

The rich diversity of each person's face images captured wildly at all ages makes it extremely difficult for machines to clean mislabeled faces effectively without sacrificing data diversity. In view of this, we propose a three-stage graph-based method to automatically clean noisy labels in extremely large face datasets by detecting communities inside the face similarity graph. As each person is represented by multiple communities, which provides fine-grained representations for various faces, our cleaning results have both high cleanness and rich data diversity. With our method, we obtain a clean version of the original “MS-Celeb-1M” database named “C-MS-Celeb” (containing 6,464,016 images of 94,682 celebrities) where approximately 97.3% of the images are correctly labeled. As far as we know, C-MS-Celeb is the largest clean face dataset that is publicly available, which will benefit face recognition groups, especially in academia. Extensive experiments show that, by training with our cleaned database, the performance of face recognition models can be significantly improved. Trained with the C-MS-Celeb dataset, our single-net Inception-ResNet-v1 model achieves 99.67% at Equal Error Rate on the LFW dataset, which is comparable to other state-of-the-art results.

## Figures and Tables

**Algorithm 1:**
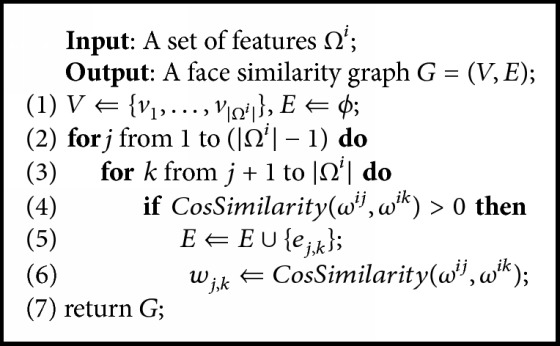
Construction of face similarity graph.

**Algorithm 2:**
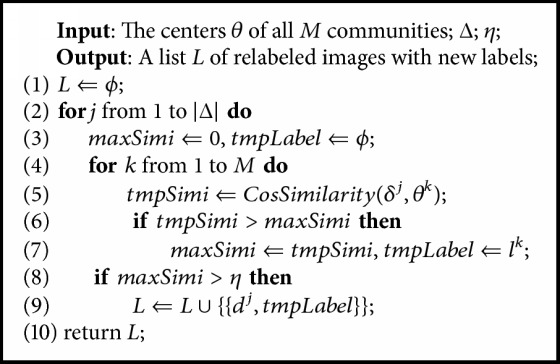
Relabeling previously deleted images.

**Table 1:** Comparison with other methods in terms of cleaning MS-Celeb-1M. Ours is able to achieve high cleanness, rich data diversity, and large data scale at the same time.

Method	*k*	*ρ*	*ζ*	Cleanness	Diversity	#Subject	#Image
Ours	-	5	-	93.6%	0.5811	97,646	7,244,505
	-	**10**	-	**97.2%**	**0.5513**	**91,180**	**6,024,931**
	-	15	-	98.3%	0.5449	84,297	5,738,961
	-	20	-	98.8%	0.5293	78,399	5,082,658

MSM	-	-	-	98.9%	0.4843	99,892	2,207,688

KCM	5	5	-	68.2%	0.7231	99,544	7,644,424
		10	-	70.1%	0.7047	99,479	7,177,587
		15	-	73.2%	0.6763	99,426	6,291,849
		20	-	74.0%	0.6386	99,384	5,119,874
	7	5	-	68.0%	0.7169	99,304	7,500,139
		10	-	72.0%	0.6816	99,227	6,428,188
		15	-	77.5%	0.6334	99,145	4,892,615
		20	-	82.9%	0.5674	94,812	3,359,597

FPR	-	-	0.2	67.7%	0.6603	99,892	6,225,585
	-	-	0.3	69.0%	0.6319	99,892	5,458,018
	-	-	0.4	75.1%	0.6035	99,892	4,679,810
	-	-	0.5	80.8%	0.5746	99,892	3,890,961
	-	-	0.55	79.5%	0.5610	99,892	3,526,922
	-	-	0.6	80.2%	0.5453	99,892	3,133,376

LCNN	-	-	-	98.8%	0.5062	79,077	5,049,824

None	-	-	-	61.1%	0.7277	99,892	8,456,240

**Table 2:** Performance (in %) of the Inception-ResNet-v1 face recognition model trained with 5 different cleaning results.

Model	Training data	#Image	Pairwise verification		Close-set identification	Open-set identification	
			Acc	VR@FAR = 0.1%	Rank-1	DIR@FAR = 1%	DIR@FAR = 0.1%
*B*_1_	LCNN result	5,049,824	99.63	99.43	98.06	94.01	82.08

*A*_1_	MS-Celeb-1M	8,456,240	99.40	97.97	96.50	86.36	59.31
*A*_2_	MS-C-1-noR	4,269,900	99.53	99.13	98.03	93.34	74.88
*A*_3_	MS-C-1	6,024,931	99.60	99.30	98.13	93.73	81.66
*A*_4_	*C-MS-Celeb*	*6,464,016*	*99.67*	*99.53*	*98.31*	*95.40*	*86.31*

**Table 3:** Comparison with other state-of-the-art results on the LFW benchmark (performance in %).

	#Net	Pairwise verification		Close-set identification	Open-set identification	
		Acc	VR@FAR = 0.1%	Rank 1	DIR@FAR = 1%	DIR@FAR = 0.1%
DeepID2+	25	99.47	-	95.00	80.70	-
Face++	4	99.50	-	-	-	-
DeepID3	25	99.53	-	96.00	81.40	-
IDL Single Model	7	99.68	99.11	97.60	94.12	89.08
*IDL Ensemble Model*	*70*	*99.77*	*99.41*	*98.03*	*95.80*	*92.09*

DeepFace	1	97.35	-	64.90	44.50	-
WebFace	1	97.73	80.26	-	-	28.90
Facebook-WST Fusion	1	98.37	-	82.50	61.90	-
CenterLoss	1	98.70	-	94.05	69.97	-
Light CNN-29	1	99.33	-	97.33	93.62	-
FaceNet	1	99.63	-	-	-	-
*Ours*	*1*	*99.67*	*99.53*	*98.31*	*95.40*	*86.31*

**Table 4:** Comparison with other state-of-the-art results on the LFW BLUFR benchmark (performance in %).

	VR@ FAR = 0.1%	D1R@FAR = 1%
HighDimLBP	41.66	18.07
WebFace	80.26	28.90
CenterLoss	93.35	67.86
Light CNN-29	98.71	90.42
*Ours*	*99.59*	*94.35*

## Supplementary Materials

Supplementary Materials This includes the final database cleaning result in our paper: the C-MS-Celeb dataset. The file list of the C-MS-Celeb includes the two TXT files in clean_list.7z: “clean_list_128Vec_WT051_P010.txt” and “relabel_list_128Vec_T058.” “clean_list_128Vec_WT051_P010.txt” contains the path of all cleaning results in Stage 2. “relabel_list_128Vec_T058.txt” contains the path of all relabeling results in Stage 3. For both files, the first column is the identity label of the image and the second column is the path of the image file. In order to use this largest clean dataset, one needs firstly to download the MS-Celeb-1M dataset and then copy the images according to the path in both TXT files. The raw MS-Celeb-1M dataset can be downloaded from the following website: https://www.microsoft.com/en-us/research/project/ms-celeb-1m-challenge-recognizing-one-million-celebrities-real-world/.
